# Polygoni Multiflori Radix Preparat Delays Skin Aging by Inducing Mitophagy

**DOI:** 10.1155/2021/5847153

**Published:** 2021-01-13

**Authors:** Xiang Liu, Chunyan Yang, Ying Deng, Ping Liu, Hongqiu Yang, Xiaoshuang Du, Yu Du

**Affiliations:** ^1^Medical Cosmetology Center, Affiliated Hospital of Traditional Chinese Medicine, Southwest Medical University, Luzhou, Sichuan 64600, China; ^2^Department of Dermatology, Affiliated Hospital of Traditional Chinese Medicine, Southwest Medical University, Luzhou, Sichuan 64600, China

## Abstract

**Background:**

As the skin is the largest organ of the human body, it is aging inevitably and produces cosmetic and psychological problems, and even disease. Therefore, the molecular mechanisms related to the prevention of skin aging need to be further explored.

**Methods:**

Aging models were constructed by D-galactose. Mice were administrated with polygoni multiflori radix preparat (PMRP), PMRP and 3-methyladenine, or PMRP and rapamycin intragastrically. The apparent and viscera index of aged rats was measured. Then, the physicochemical property, antioxidant ability, histological structure, mitochondrial membrane potential, ATP and ROS levels, and mitophagy of aged skins were determined. Finally, the expression of PINK1, Parkin, P62, and LC3II/I; apoptosis-related proteins; and the percentage of apoptotic cells were measured.

**Results:**

PMRP relieved skin aging with reducing of thymus index, improvement of pathological damage and histological structure, increase of the expression area of fibrous tissue, the ratio of type I to type III collagen, and antioxidant ability of aged skins. Importantly, PMRP also improved mitochondrial dysfunction with an increase in the content of mitochondrial membrane potential and ATP and a decrease of ROS levels. Moreover, mitophagy was enhanced with the treatment of PMRP when observed using electron microscopy, and the expression of PINK1, Parkin, and LC3I/II was increased with PMRP treatment but P62 expression was decreased. Meanwhile, PMRP alleviated apoptosis with a decrease of apoptotic cell and the expression of Cleaved-cas3, Bax, Cyt-c, AIF, and Smac as well as an increase of Bcl-2 expression.

**Conclusion:**

The results demonstrated that the polygoni multiflori radix preparata may delay skin aging by inducing mitophagy.

## 1. Introduction

The skin is the most voluminous organ with the largest area of contact with the external environment in the human body [1]. It provides physical parcloses to the external environment, prevents loss of excessive water, maintains physiological homeostasis, and also has certain cosmetic effects [2]. However, skin aging is an inescapable physiological process. With the development of science and technology and the improvement of people's living standards, many people, especially women, spend a large part of their daily expenses on cosmetics and medicines to treat and prevent skin aging. Therefore, it is urgent to explore the molecular mechanisms related to the prevention of skin aging.

Mitochondria are the major organelles for energy production of eukaryotic cells to grow and survive in an aerobic environment. Their functions are multifaceted including generation of adenosine triphosphate [3], regulation of reactive oxygen species (ROS) [4], transduction of calcium signaling [5], participation in programmed cell death [6], and tumorigenesis [7]. Hence, mitochondria play an important role in maintaining intracellular homeostasis, and their dysfunction can lead to cell dysfunction and even cell death [8]. Several mechanisms are involved in the prevention of the accumulation of damaged mitochondria [9]. Therein, it is nonnegligible for mitophagy to reduce mitochondrial damages and maintain homeostasis [10]. Mitophagy is a kind of autophagy that selectively removes damaged or excess mitochondria and transmits them to the lysosome for degradation [11]. Under the action of external stimuli such as ROS and nutritional deficiency, the mitochondrial membrane is depolarized to cause mitochondrial damages, and then, damaged mitochondria are selectively encapsulated into autophagosomes and fused with lysosomes to complete the degradation and removal of damaged mitochondria [12]. Mitophagy plays a key regulatory role in maintaining mitochondrial energy homeostasis, quality control, and cellular immunity [12–14] and is highly associated with apoptosis [15] and necroptosis [13]. Moreover, increasing evidence indicates that mitophagy is also correlated with aging in different organs of the organism, such as the brains [16], hearts [17], and muscles [18]. However, the effect of mitophagy on skin aging is still little known. Thus, the effect of mitophagy on skin aging should be clearly elucidated.

*Polygonum multiflorum* Thunb. (Polygoni Multiflori Radix) is one of traditional Chinese medicines and has exhibited antioxidative activities [19], antiaging effects [20], anti-inflammatory effects [21], and antitumor effects [22]. Moreover, a previous study has reported that *P. multiflorum* can alleviate mitochondrial dysfunction *in vitro* [23]. Thus, we speculate that *P. multiflorum* may play a role in delaying skin aging by regulating mitophagy. However, liver injury induced by *P. multiflorum* has been an increasing concern in recent years [24]. Therefore, in this present study, we explore polygoni multiflori radix preparata (PMRP), which is the processed product of *P. multiflorum*, and its efficacy is better and its toxicity is lower than that of *P. multiflorum*, which act on delaying skin aging by regulating mitophagy. The results of this study will provide new ideas for the function of *P. multiflorum*.

## 2. Materials and Methods

### 2.1. Reagents

D-Galactose, 3-methyladenine, and rapamycin were purchased from Sigma-Aldrich (Merck KGaA, Darmstadt, Germany). Polygoni multiflori radix preparata was purchased in the pharmacy of the Affiliated Hospital of Chengdu University of Traditional Chinese Medicine. The remaining reagents used in this study were commercially available and of analytical purity.

### 2.2. Animal

Two-month-old male rats (~200 g) were purchased from Chengdu Dashuo Biological Technology Co., Ltd. All mice were fed with a standard diet and had free access to water. All the animal experiments complied with ethical standards as determined by the ethical committee of the West China Hospital of Sichuan University (2020035A). After adaptive rearing for one week, 50 rats were randomly divided into a control group (Control), aging model group (Model), PMRP intragastrical administration group (PMRP), PMRP and 3-methyladenine intragastrical administration group (PMRP+3-AM), and PMRP and rapamycin intragastrical administration group (PMRP+RAPA) (*n* = 10). The Model, PMRP, PMRP+3-AM, and PMRP+RAPA group were subcutaneously injected with D-galactose (200 mg·kg^−1^) to build the subacute senescence model, and the Control group was injected equivalent saline. The injection was continuous for 8 weeks. After 4 weeks of modeling, the PMRP, PMRP+3-AM, and PMRP+RAPA group used PMRP (40 g·kg^−1^), PMRP and 3-AM (7 mg·kg^−1^), and PMRP and RAPA (0.5 mg·kg^−1^) intragastrical injection, respectively. Meanwhile, the Control and Model groups used the same amount of saline. After continuous intragastrical treatment for 10 weeks, rats were anesthetized with 0.3% pentobarbital sodium (0.1 ml/10 g) and sacrificed to collect organs and skin tissues for further analysis.

### 2.3. Histological Analysis

Skin tissues were fixed in 10% formalin for 24 h and stained with hematoxylin and eosin (H&E), Masson's trichrome, and Sirius red stain, respectively. The stained sections were analyzed using the digital trinocular camera microscope (BA400Digital, McAudi Industry Group Co., Ltd.) and image analysis software Image-Pro Plus 6.0 (Media Cybernetics, USA).

### 2.4. Detection of Antioxidant of Skins

Skin tissues were homogenized on ice and centrifuged with 3500 rpm, and the sample supernatant was collected for the detection of the activity of SOD, MDA, ALT, AST, and GSH-Px using SOD (A001-1-2), MDA (A003-1-2), ALT (C009-2-1), AST (C010-2-1), and GSH-Px (A005-1-2) assay kits (Nanjing Jiancheng Bioengineering Institute, China) according to the instructions. SOD assay involves a xanthine-xanthine oxidase system that reacts with 2-(4-iodophenyl)-3-(4-nitrophenol-5-phenlyltet-razolium chloride) to form a red formazan dye at an absorbance at 550 nm and produces superoxide ions. The protein concentration was determined using a BCA protein assay kit (Pierce Chemical Co.), with one unit of SOD defined as the amount of SOD inhibiting the rate of reaction by 50% at 25°C. MDA detection is based on the spectrophotometric measurement of color produced during the MDA reaction with TBA. MDA concentrations were calculated through the absorbance of TBA reactive substances (TBARS) at 532 nm. ALT concentrations were calculated through the absorbance of NADH at 340 nm at 37°C. AST concentrations were calculated through a microplate reader at 510 nm at room temperature. GSH-Px concentrations were calculated through spectrophotometric at 412 nm at room temperature.

### 2.5. Measurement of Histological Structure of Skins

We first calculated the total skin thickness with the equation: *d* = (*m*/*ρ*)/*S*, and *d* is the skin thickness (mm), *m* is the skin mass (mg), *ρ* is the skin density (1.04 g∙mm^−3^), and *S* is the skin area (mm^2^). Next, the hydroxyproline level of the skins was determined. Skin tissues were homogenized and then hydrolyzed at 105°C for 16 h. The hydroxyproline assay kit (Quickzyme Bioscience, Leiden, Netherlands) was used to measure the hydroxyproline content according to the operating instructions.

### 2.6. Detection of Mitochondrial Membrane Potential

The mitochondria of skins were isolated with the Mitochondria Extraction Kit (G006-1-1, Nanjing Jiancheng Bioengineering Institute, China) according to the operating instructions, stained using the mitochondrial membrane potential detection kit (JC-1) (G009-1-3, Nanjing Jiancheng Bioengineering Institute, China) according to operating instructions, and analyzed by flow cytometry (BD FACSVerse) with 5 *μ*l Annexin V-PE (Sigma, Germany) and 5 *μ*l FITC (Sigma, Germany) staining.

### 2.7. Detection of Mitochondrial ATP

The mitochondria of skins were isolated with the Mitochondria Extraction Kit (G006-1-1, Nanjing Jiancheng Bioengineering Institute, China) according to the operating instructions, and then, the mitochondrial ATP level was detected using the ATPase kit (G016-1-2, Nanjing Jiancheng Bioengineering Institute, China) according to the operating instructions.

### 2.8. Detection of ROS of the Skin

Reactive Oxygen Species Assay Kit (S0033, KeyGen BioTECH, China) was used to measure the level of ROS of skins according to operating instructions.

### 2.9. Electron Microscopy

Skin tissues were fixed in 3% glutaraldehyde and 1% osmium tetroxide and cut on an ultramicrotome. Then, sections were stained with 1% uranyl acetate and 0.5% lead citrate successively. The results were observed using the JEM-1400PLUS transmission electron microscope.

### 2.10. SDS-PAGE and Western Blot Analysis

The total protein from skin tissues was extracted using a Total Protein Extraction Kit (BC3711, Solarbio), and the protein concentration was quantified using the BCA protein quantification kit (ab102536, Abcam) according to the operating instructions. Protein samples were separated by 12% SDS-PAGE and then transferred to a PVDF membrane electrically at 150 V for 4 h. After preblocking with TBST (Sigma Aldrich, America) (containing 3% BSA) for 1 h at room temperature, the membrane was incubated with the primary antibody (1 : 1000 rabbit anti-PINK-1, 1 : 1000 rabbit anti-Parkin, 1 : 2000 rabbit anti-P62, 1 : 1000 rabbit anti-LC3 I/II, 1 : 500 rabbit anti-caspase-3, 1 : 1000 rabbit anti-Cleaved-cas3, 1 : 1000 rabbit anti-Bax, 1 : 1000 rabbit anti-Cyt-c, 1 : 1000 rabbit anti-AIF, 1 : 1000 rabbit anti-Smac, 1 : 500 rabbit anti-Bcl-2, 1 : 100,000 rabbit polyclonal anti-*β*-actin) at 4°C overnight. After washing three times with TBS, the membrane was incubated with goat-anti-rabbit IgG (H+L)-HRP (ab6721, Abcam) diluted 1 : 5,000 in TBST (containing 3% BSA) for 1 h. The visualization of the reaction was carried out using DAB (Sigma, USA) for 5 to 15 min and stopped by rinsing with distilled water.

### 2.11. TUNEL Staining

TUNEL staining was performed by the *in situ* cell death detection kit POD (Roche, America) according to the operating instructions. The sections were mounted using a mounting medium, antifading (with DAPI) (S2110, Solarbio) after washed 3 times in 0.1 M PBS. Six random nonoverlapping fields (×400) were selected to analyze TUNEL-positive cells quantificationally.

### 2.12. Statistical Analysis

All the results were shown as the means ± SD. Differences between multiple groups were analyzed using one-way analysis of variance and Duncan's test using the SPSS 19.0 package (SPSS Inc., Chicago, IL, USA). The differences were considered statistically nonsignificant and significant when *p* > 0.05 and *p* < 0.05, respectively.

## 3. Results

### 3.1. PMRP Influences the Apparent Index of Aged Rats

**Figure 1:**
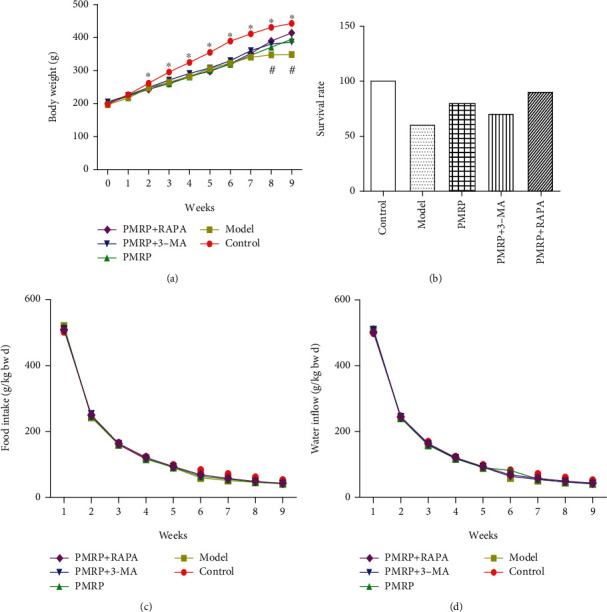
Effect of PMRP on the apparent index of the aged rat skins. Rats were administrated PMRP, PMRP+3-AM, PMRP+RAPA, or saline for 10 weeks intragastrically, and the body weight (a), food intake and water inflow (c and d), and mortality (b) were recorded. Food intake (g/kg bw d) = (additional amount of feed − remaining amount of feed)/total weight of mice/days, water intake (g/kg bw d) = (water addition amount − water remaining amount)/total weight of mice/days, mortality (%) = (initial quantity − final quantity)/initial quantity. The means ± SD of four independent samples in body weight are shown. The expression levels among the different groups were determined by one-way ANOVA. The differences are significantly different (*p* < 0.05) showing with ^∗^ or ^#^.

We first detected the apparent index of aged rat skins. Body weight can measure the health of the body to a certain extent, in which too high or too low both can cause disease and even affect lifespan. Body weight in the Control group was significantly higher than that in the Model, PMRP, PMRP+3-AM, and PMRP+RAPA group from week 2 to 9, while that in the Model group was significantly lower than that in the other four groups at weeks 8 and 9 (Figure 1(a)). The results suggested that PRMP can improve weight loss caused by aging in the later stages of the trial. However, food intake and water inflow were no different among the five groups (Figures 1(c) and 1(d)). Additionally, mortality in the Model group was the highest, and PRMP could reduce mortality caused by aging (Figure 1(b)). Briefly, the results indicated that PRMP improved weight loss and mortality caused by aging.

### 3.2. PMRP Acts on the Viscera Index of the Aged Rats

**Figure 2:**
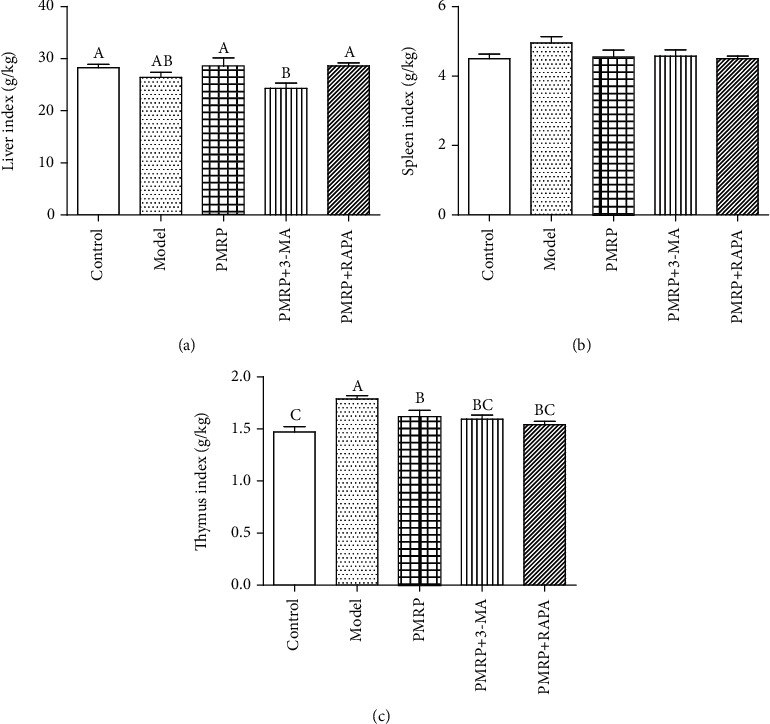
Effect of PMRP on the viscera index of the aged rat skins. After continuous intragastrical treatment for 10 weeks, rats were anesthetized with 0.3% pentobarbital sodium (0.1 ml/10 g) and sacrificed to collect liver, spleen, and thymus for viscera index analysis. The viscera index was calculated with the equation: the viscera index = organ weight (g)/body weight (g) × 100%. The means ± SD of four independent samples are shown. The expression levels among the different groups were determined by one-way ANOVA. The difference is significantly different (*p* < 0.05) where the letters over the bars are different.

Then, the effect of PMRP on the liver, spleen, and thymus index was assessed. The results showed that there was no significant difference in both the liver and spleen index between the Model group and Control group, while the thymus index in the Model group was significantly higher than that in the Control group (Figures 2(a)–2(c)), indicating that the D-galactose model may cause swelling of the thymus. However, PMRP treatment significantly decreased the thymus index compared with the Model group suggesting that PMRP could inhibit the swelling of the thymus (Figure 2(c)). Since thymus aging is one of the most important body reactions during aging, PMRP might have an effect of delaying aging. Furthermore, inhibition or activation of mitophagy had no effect on the spleen and thymus index, while inhibition of mitophagy significantly declined the liver index. In brief, PMRP might improve the aging skin in rats.

### 3.3. PMRP Functioned on the Physicochemical Property of the Aged Rat Skins

**Figure 3:**
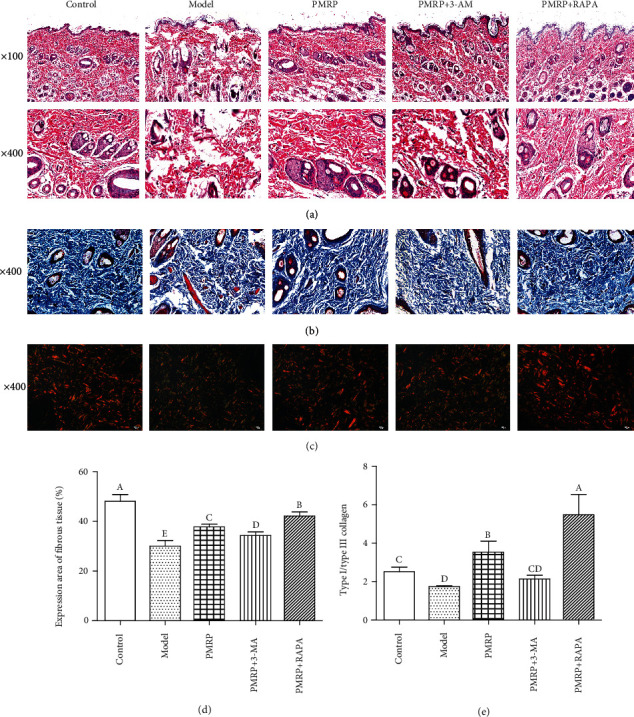
Effect of PMRP on the physicochemical property of the aged rat skins. The physicochemical property of the aged rat skins was detected among different groups using H&E (a), Masson's trichrome (b), and Sirius red stain (c). The percentage of the expression area of fibrous tissue (d) and the ratio of type I to type III collagen of the skin tissues (e) were also detected, respectively. The means ± SD of three independent samples are shown. The expression levels among the different groups were determined by one-way ANOVA. The difference is significantly different (*p* < 0.05) where the letters over the bars are different.

Next, the physicochemical property of the aged rat skins was detected among different groups using H&E, Masson's trichrome, and Sirius red stain. First, the pathological damage of the skin epithelial cells was observed using H&E staining. The pathological changes of incomplete epidermal structure, fewer and thinner dermal collagen fibers, a disordered arrangement of dermal collagen fibers, and infiltration of dermal inflammatory cells, were observed in the Model group compared with the Control group, while the pathological changes of the Model group were best relieved in the PMRP+RAPA group, followed by the PMRP group and then the PMRP+3-AM group (Figure 3(a)). Subsequently, since collagen fibers are green using Masson staining, the distribution of skin collagen was determined using Masson stain. Compared with the Control group, the percentage of the expression area of the fibrous tissue of the skin tissue in the Model group was significantly reduced, suggesting that the content of collagen fibers of the skin tissue in the Model group was significantly reduced (Figures 3(b) and 3(d)). However, the percentage of the expression area of fibrous tissue of the skin tissue in the PMRP, PMRP+3-AM, and PMRP+RAPA groups was significantly higher than that in the Model group (Figures 3(b) and 3(d)). Reduced production of collagen types I and III is a characteristic of chronologically aged skin [25]. Sirius red can combine collagen fibers of different diameters, so it can distinguish type I (yellow or red) and type III collagen (green). Hence, the proportion of collagen I and III was detected using Sirius red staining. Similarly, the ratio of type I to type III collagen of the skin tissue in the Model group was significantly decreased compared with the Control group, while that in PMRP and PMRP+RAPA groups increased significantly compared with the Model group (Figures 3(c) and 3(e)). Taken together, these results suggested that the animal model caused by D-galactose present aging skin and the aging skin could be improved by PMRP.

### 3.4. PMRP Enhanced the Antioxidant Ability of the Aged Rat Skins

**Figure 4:**
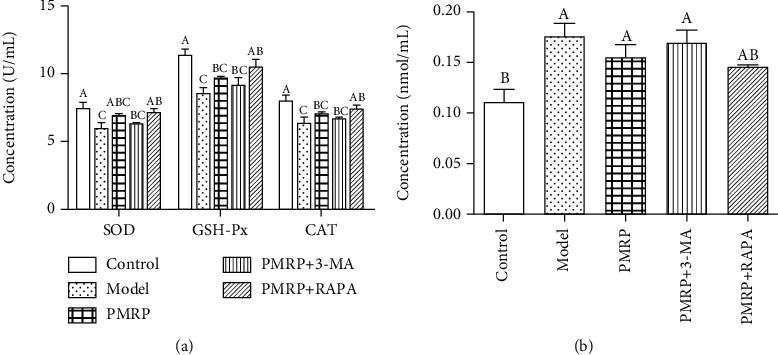
PMRP increased the antioxidant ability of the aged rat skins. The level of SOD, GSH-Px, CAT (a), and MDA (b) of the aged rat skins was measured among different groups. The means ± SD of three independent samples are shown. The expression levels among the different groups were determined by one-way ANOVA. The difference is significantly different (*p* < 0.05) where the letters over the bars are different.

The generation and elimination of ROS is an important factor leading to aging [26], and aerobic organisms inevitably produce ROS during oxygen metabolism. However, organisms have also evolved enzymatic antioxidant defense systems. For example, SOD can promote the conversion of superoxide anions to hydrogen peroxide, and GSH-Px can catalyze glutathione reaction to inhibit the production of hydrogen peroxide and lipid peroxides [27]. Hence, we measured the level of SOD, GSH-Px, MDA, and CAT in aging skins. The results showed that the level of SOD, GSH-Px, and CAT in the Model group was significantly lower than that in the Control group (Figure 4(a)). The SOD, GSH-Px, and CAT levels in the PMRP and PMRP+3-AM groups were higher than those in the Model group, while those in the PMRP+RAPA group were significantly higher than those in the Model group (Figure 4(a)). The level of MDA in the Model group was significantly higher than that in the Control group (Figure 4(b)). The MDA level in the PMRP, PMRP+3-AM, and PMRP+RAPA groups was lower than that in the Model group (Figure 4(b)). Briefly, the results above indicated that PMRP could enhance the antioxidant ability.

### 3.5. PMRP Improved the Histological Structure of the Aged Rat Skins

**Figure 5:**
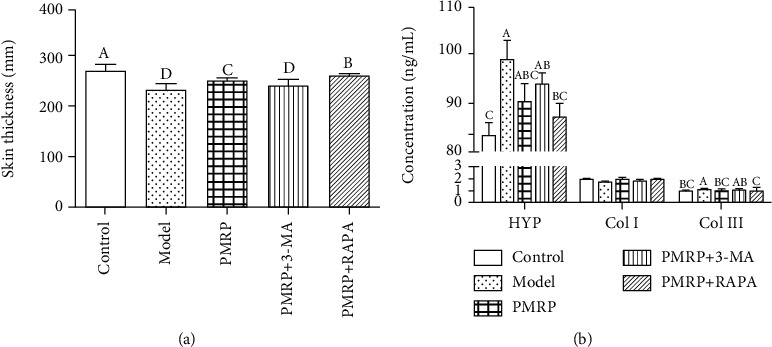
PMRP improved the histological structure of the aged rat skins. The total thickness of the skin (a) and the level of HYP, collagen I, and collagen III (b) of the aged rat skins were determined among different groups. The means ± SD of three independent samples are shown. The expression levels among the different groups were determined by one-way ANOVA. The difference is significantly different (*p* < 0.05) where the letters over the bars are different.

The natural aging of the skin causes the thickness of the dermis and epidermis to become thin, so the total thickness of the skin also becomes thin. The total thickness of the skin in the Model group was significantly decreased compared with the Control group, while that in the PMRP and PMRP+RAPA groups increased significantly compared with the Model group (Figure 5(a)). The results suggested that PMRP could improve the total thickness of the skin. Meanwhile, the content of collagen III was significantly higher than that in the Control group, while that in the PMRP and PMRP+RAPA groups was significantly lower than that in the Model group. The collagen I level was decreased compared with the Control group, while that in the PMRP, PMRP+3-AM, and PMRP+RAPA groups increased compared with the Model group (Figure 5(b)). Moreover, the level of hydroxyproline (HYP) was significantly higher than that in the Control group, while that in the PMRP, PMRP+3-AM, and PMRP+RAPA groups was lower than that in the Model group (Figure 5(b)). Thus, these data indicated that PMRP improved the histological structure of the aged rat skins.

### 3.6. PMRP Acted on the Mitochondrial Function of the Aged Rat Skins

**Figure 6:**
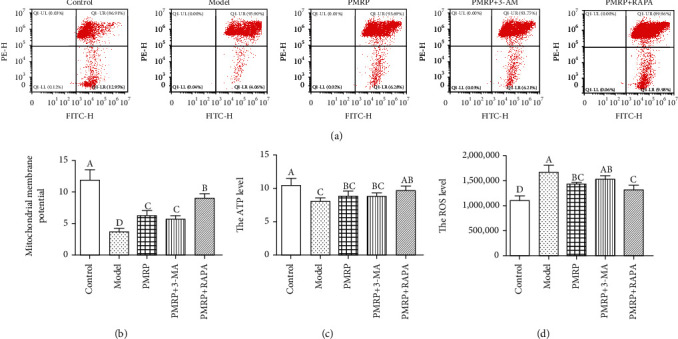
PMRP improved the mitochondrial dysfunction in the aged rat skins. The mitochondrial membrane potential was determined using flow cytometry with Annexin V-PE and FITC stain (a). The ATP (b) and ROS (c) levels of mitochondrial in the aged rat skins also were measured. The means ± SD of three independent samples are shown. The expression levels among the different groups were determined by one-way ANOVA. The difference is significantly different (*p* < 0.05) where the letters over the bars are different.

All the results above suggested that PMRP could improve the aging skins, but its underlying mechanism is still unclear. A previous study showed that mitochondrial dysfunction is a key pathological feature leading to aging [3]. Hence, we first assessed the mitochondrial function of the aged rat skins. The results revealed that the mitochondrial membrane potential in the Model group was significantly decreased compared with the Control group, while that in the PMRP, PMRP+3-AM, and PMRP+RAPA groups increased significantly compared with the Model group (Figures 6(a) and 6(b)). Consistently, the ATP levels in the mitochondria in the Model group were significantly lower than those in the Control group, while those in the PMRP and PMRP+3-AM groups were higher and in the PMRP+RAPA group significantly higher than those in the Model group (Figure 6(c)). However, the ROS levels in the mitochondria were significantly enhanced compared with the Control group, while those in the PMRP and PMRP+RAPA groups declined significantly compared with the Model group (Figure 6(d)). Therefore, PMRP improved the mitochondrial dysfunction in the aged rat skins.

### 3.7. PMRP Promoted Mitophagy of the Aged Rat Skins

**Figure 7:**
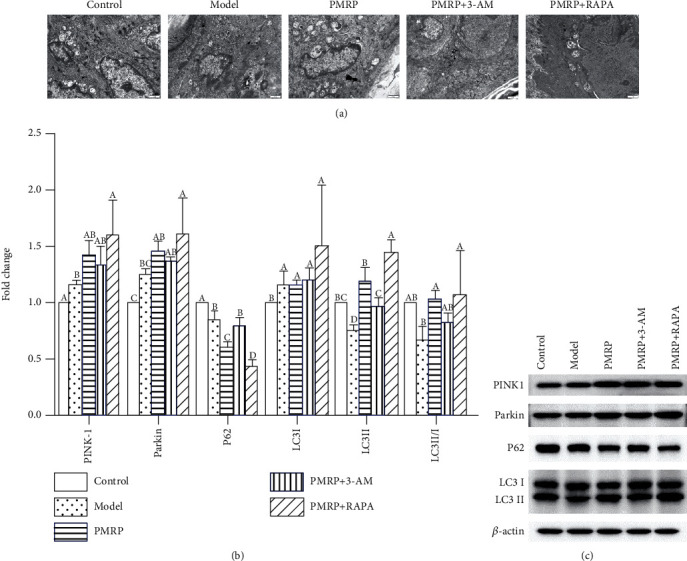
PMRP promoted mitophagy of the aged rat skins. (a) The morphological structure and formation of mitophagy in the aged rat skin cells were observed using electron microscopy (b and c). The expression of mitophagy-related proteins, such as PINK1, Parkin, P62, and LC3II/I, was detected using western blotting. The means ± SD of three independent samples are shown. The expression levels among the different groups were determined by one-way ANOVA. The difference is significantly different (*p* < 0.05) where the letters over the bars are different.

Then, the morphological structure and formation of mitophagy in the aged rat skin cells were observed using electron microscopy. The results revealed that the swelling of mitochondria was found in the Model, PMRP, PMRP+3-AM, and PMRP+RAPA groups compared with the Control group (Figure 7(a)). Furthermore, mitophagy was observed in the Model group, and PMRP and PMRP+RAPA treatment increased mitophagy, but PMRP+3-AM treatment decreased mitophagy (Figure 7(a)). Moreover, dysfunctional mitochondria in the body are mainly cleared through mitophagy [28]. Among different pathways involved in mitophagy, the phosphatase and tensin homologue- (PTEN-) induced putative kinase 1 (PINK1) and the E3-ubiquitin ligase Parkin were the most studied pathway [28]. The expression of PINK1 and Parkin in the Model group was higher than that in the Control group, indicating that there was increasing mitophagy in the aged model induced by D-galactose (Figures 7(b) and 7(c)). Importantly, the PMRP+RAPA treatment significantly enhanced the content of mitophagy, and PMRP and PMRP+3-AM treatment augmented mitophagy level, which suggests that PMRP could promote mitophagy level in aged rat skins (Figures 7(b) and 7(c)). In the PINK1 and Parkin pathway, the adaptor protein P62 can bind to LC3 that triggers mitophagy and further degrades damaged mitochondria. LC3 is a key protein produced by autophagosomes and also a labeled protein on the autophagosome membrane. The ratio of LC3II/I reflects the number of autophagosomes formed and is also an important indicator of the autophagy extension phase [29]. The results showed that the expression of LC3II/I in the Model group was lower than that in the Control group, and the PMRP and PMRP+RAPA treatment enhanced the content of mitophagy level (Figures 7(b) and 7(c)). As a selective autophagic degradation substrate, P62 participates in the process of autophagy. It is a sign of autophagosome degradation activity and also reflects the activity of lysosomal hydrolase [30]. The P62 expression in the Model group was significantly lower than that in the Control group, and the PMRP and PMRP+RAPA treatment further significantly decreased the level of mitophagy (Figures 7(b) and 7(c)). Overall, mitophagy was enhanced by PMRP in the aged rat skins.

### 3.8. PMRP Alleviated Apoptosis of the Aged Rat Skins

**Figure 8:**
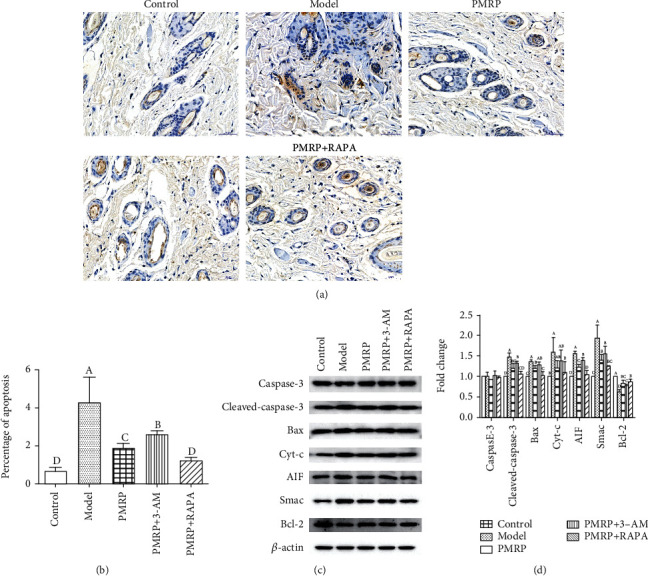
PMRP relieved apoptosis of the aged rat skins. (a and b) The percentage of apoptotic in the aged rat skin cells using TUNEL. (c and d) The expression of apoptosis-related proteins, such as caspase-3, Cleaved-cas3, Bax, Cyt-c, AIF, Smac, and Bcl-2 was detected using western blotting. The means ± SD of three independent samples are shown. The expression levels among the different groups were determined by one-way ANOVA. The difference is significantly different (*p* < 0.05) where the letters over the bars are different.

Furthermore, it has been reported that Parkin-mediated mitophagy is connecting to the regulation of cellular apoptosis [31]. Thus, we determined the apoptotic cell in the aged rat skin cells using TUNEL. The results revealed that the percentage of apoptotic cell in the Model group was significantly increased compared with the Control group, while that in the PMRP, PMRP+3-AM, and PMRP+RAPA groups decreased significantly compared with the Model group (Figures 8(a) and 8(b)), suggesting that PMRP could relieve the apoptotic cell in the aged rat skins. Additionally, the expression of proteins involved in apoptosis including caspase-3, Cleaved-cas3, Bax, Cyt-c, AIF, Smac, and Bcl-2 was also detected using western blotting. The results showed that the expression of Cleaved-cas3, Bax, Cyt-c, AIF, and Smac in the Model group was significantly higher than that in the Control group, while that in PMRP PMRP+3-AM and PMRP+RAPA groups was obviously lower than that in the Model group (Figures 8(c) and 8(d)). Meanwhile, the level of Bcl-2 in the Model group was significantly lower than that in the Control group, while that in the PMRP PMRP+3-AM and PMRP+RAPA group was significantly higher than that in the Model group (Figures 8(c) and 8(d)). There was no statistical difference in the caspase-3 expression among different groups. Taken together, the results indicated that PMRP could alleviate apoptosis.

## 4. Discussion

Aging is directly related to the life cycle and also a direct cause of the increased incidence of age-related diseases. Therefore, aging and age-related diseases are becoming one of the biggest challenges and bring a heavy financial burden to countries. More importantly, with the gradual improvement of human living standards and the rapid development of modern medicine, skin aging has received more and more attention. Therefore, huge researches need to be focused on the prevention and treatment of skin aging. In this study, we investigated the effect of PRMP on skin aging. This study revealed that PRMP can relieve skin aging by regulating autophagy.

As a commonly used animal aging model in the laboratory, the subacute aging model induced by D-galactose can simulate multiple organs aging, such as the brain [32], liver [33], kidney [34], skin [35], skeletal muscle [36], and immune system [37], thereby making this model widely used to explore the mechanism of aging and the development of antiaging drugs. In this present study, we also chose D-galactose to establish skin aging models. The results showed that in the Model group established by D-galactose, there were obvious pathological changes in the skin tissues, downregulation of collagen distributions, decrease of collagen I, increase of collagen III, decrease of antioxidant capacity, and decrease of total skin thickness, which indicated that the skin aging model induced by D-galactose was successfully constructed. Moreover, the use of polygoni multiflori radix preparat could alleviate the pathological changes in the abovementioned aging model; thus, it can be seen that polygoni multiflori radix preparat can effectively delay the aging of the skin.

Several molecular mechanisms of skin aging have been reported, including oxidative stress [38], DNA damage and gene mutation [39], shortening of the telomere [40], the role of microRNA [41], accumulation of advanced glycation end products (AGEs) [42], and aging due to inflammation [43]. Recently, more and more studies showed that mitophagy is involved in aging [28]. Electron microscopic observation revealed that the treatment of PRMP increased the autophagosomes in mitochondria, suggesting that PRMP can contribute to mitophagy of the aged skins. As the classic mitophagy signaling pathway, the PINK1 and Parkin were the most studied pathway [28]. Upon mitochondrial damage or dysfunction, the mitochondrial membrane potential decreases, prompting PINK1 to accumulate in the outer membrane of the mitochondria and combine with the outer membrane translocating enzyme (TOM) to form a 700 kD complex, thereby recruiting Parkin in the cytosol to translocate to damaged mitochondria. Due to their E3 ubiquitinase activity, mitochondrial outer membrane surface proteins such as VDAC1 and Mnf1/2 were ubiquitinated and then recruited the linker protein p62 or HDAC6 connected to the ubiquitin substrate. The linker protein binds to LC3, which degrades the damaged mitochondria by autophagy [44]. In this study, we detected the mitochondrial membrane potential using JC-1. The mitochondrial membrane potential is a prerequisite for oxidative phosphorylation of mitochondria to produce ATP and plays an important role in maintaining mitochondrial function. Studies have found that a variety of cells are accompanied by a decrease in mitochondrial membrane potential levels when mitochondrial damage or dysfunction occurs [45]. The level of mitochondrial membrane potential and ATP in the Model group was significantly decreased, indicating that mitochondrial damage or dysfunction was in aging skins. Thus, the expression of Parkin and PINK-1 in the Model group increased significantly, indicating that mitophagy appeared in the aging skin to relieve aging. Moreover, the use of PRMP could further increase the expression of Parkin and PINK-1, indicating that PRMP could promote mitophagy to delay aging skin.

Additionally, it has been reported that Parkin-mediated mitophagy is related to the regulation of cellular apoptosis [31]. The change of mitochondrial membrane potential can promote the release of Cyt-c, Smac, and AIF from mitochondrion into the cytosol, resulting in the activation of caspase and apoptosis [46]. Besides, the Bcl-2 family proteins play a major role in the apoptosis process as a modulator of outer mitochondrial membrane integrity [47, 48]. Among the Bcl-2 family proteins, the antiapoptotic proteins such as Bcl-2 stabilize the barrier function of the mitochondrial membrane, while the proapoptotic members such as Bax destabilize this function [49, 50]. The level of the apoptotic cell and apoptosis-related proteins (caspase-3, cleaved-caspase3, Cyt-c, Smac, AIF, Bax, and Bcl-2) were reduced with treatment of PRMP when determined using TUNEL and western blotting, respectively, suggesting that PRMP treatment can improve the apoptosis.

## 5. Conclusion

In conclusion, the results from our study indicated the PRMP delay skin aging by regulating mitophagy. Our finding can further understand the function of *Polygonum multiflorum* and also lay a foundation of the mitophagy being a target to delay aging skin.

## Data Availability

The datasets used or analyzed during the current study are available from the corresponding author.
